# The Relationship Between Patient Demographics, Tear Locations, and Operative Techniques on the Surgical Treatment of Acute Achilles Tendon Ruptures

**DOI:** 10.7759/cureus.28300

**Published:** 2022-08-23

**Authors:** Josh Giordano, Matthew Partan, Cesar Iturriaga, Joseph Granata, Gus Katsigiorgis, Randy Cohn, Adam Bitterman

**Affiliations:** 1 Orthopaedics, Northwell Health, Huntington, USA; 2 Orthopaedic Surgery, Northwell Health, Huntington, USA; 3 Orthopaedics, New York Institute of Technology (NYIT) College of Osteopathic Medicine, Old Westbury, USA; 4 Orthopaedics, North Shore Long Island Jewish Health Systems, Huntington, USA

**Keywords:** foot and ankle, achilles tendon rupture, age, bmi, achilles tendon

## Abstract

Introduction

Achilles tendon ruptures (ATRs) have classically been thought to affect the middle-aged “weekend warrior” participating in basketball, volleyball, soccer, or any ground sport; however, with a more active elderly population, these tears are becoming more common in older patients. We sought to examine the role of demographics, tear location, and operative technique for acute Achilles tendon ruptures treated surgically.

Methods

A retrospective query was performed to identify patients who presented with Achilles tendon ruptures. Inclusion data were primary end-to-end repairs, augmented repairs with flexor hallucis longus (FHL) transfers, augmented repairs with graft, augmented repairs with both FHL transfer and graft use, isolated FHL transfers, and revision Achilles tendon procedures. Demographics and tear locations were collected and analyzed.

Results

Midsubstance tears were the most common tear location occurring in 237 of 286 (82.9%) patients. Distal insertional tears of the Achilles tendon were treated in 35 (12.2%) patients, while 14 (4.9%) patients had a more proximal tear located at the myotendinous junction. Older patients (average age: 53.3±12.5) had significantly more distal insertional tears (p<0.001), while younger patients (average age: 35.1±7.4) presented with significantly more tears at the myotendinous junction (p<0.001). The average BMI was significantly higher (average BMI: 32.2±6.6; p<0.001) in patients with distal insertional tears compared to midsubstance and proximal tears (28.5±4.6 and 28.5±5.3, respectively). There was a higher percentage of diabetic patients who underwent operative treatment for distal insertional tears (20%) compared to midsubstance tears (7.2%).

Conclusion

The findings of our study suggest that a subset of patients, particularly those with advanced age and higher BMI, is more likely to present with a distal Achilles tendon rupture. Additionally, patients in our series who had distal tears more commonly required an augmented repair technique. Our results highlight the need for future research to further define the relationship between increasing age and higher BMI patients sustaining distal tears more often than midsubstance tears.

## Introduction

The Achilles tendon is the most frequently ruptured tendon with an increasing incidence over the past few decades [1-4]. Achilles tendon ruptures (ATRs) have classically been thought to affect the middle-aged “weekend warrior” participating in basketball, volleyball, soccer, or any ground sport that requires speed and agility. However, with a more active elderly population, these tears are becoming more common in older patients [5-8]. Loss of Achilles function leads to loss of plantar flexion strength, weakness, fatigue, limp, inability to run, heel rise, and difficulty playing sports and climbing stairs [3,9-11]. Delay in treatment, whether operative or nonoperative, has detrimental effects on the final outcomes [12-17].

The optimal treatment of ATRs currently remains a topic of debate [9,18-20]. The zone of injury becomes an important aspect of determining the optimal surgical technique. Although ATRs most commonly occur at the midsubstance region of the tendon (2-6 cm from the calcaneal insertion), a subset of tears does rupture outside of this zone, making fixation potentially more difficult. It is recognized that distal injuries, outside of the midsubstance parameters, make fixation strategies particularly difficult. The difficulty results from the little or poor quality of the tendinous tissue remaining distally following rupture distally [21]. If enough tissue is present, reinsertion of the Achilles using a transcalcaneal suture technique can be accomplished [22]. However, if the remaining tendon is of poor quality, augmentation may be necessary. In a series of 26 patients, Schipper et al. treated acute insertional Achilles tendon avulsions with suture anchors and FHL tendon transfer if intraoperatively more than 50% of the width of the insertional tendon was detached [23]. In another study, Maffulli et al. described a technique using an ipsilateral free semitendinosus graft with interference screw fixation for acute insertional Achilles ruptures [24]. The debate over proper repair technique is ongoing, but it is clear that distal ruptures of the Achilles tendon can present difficulty to the operating surgeon.

Various risk factors have been examined in the literature as increased risk for ATRs. Increasing age is a known demographic risk factor for ATR as it leads to chronic degeneration of the Achilles tendon. Such degeneration is coupled with decreased blood flow and a decrease in the tensile strength of collagen within the tendon [25]. Collagen is responsible for resisting the tensile forces applied to the tendon. With aging, collagen becomes stiffer, has reduced tensile strength, and develops an increase in the cross-linking of the tropocollagen molecules. These factors make aged tendons more likely to tear [26]. Increased BMI (BMI ≥ 30 kg/m^2^) may also play a role in ATRs. Obesity has been shown to increase the risk of tendinopathy, which in turn puts the tendon at risk for rupture [27-30]. Although the above risk factors have been implicated in higher-risk patient populations, inclusive analysis of the above has yet to be examined.

We sought to determine if a relationship between demographics and tear location exists and, if so, how surgical technique is affected by tear location and demographics in the operatively treated Achilles tendon rupture. We hypothesized that there would be no difference between age, BMI, and tear location.

## Materials and methods

Following institutional board approval, a retrospective query of our institution’s electronic medical record was performed to identify all patients who presented to our multicenter institution from January 1, 2017, to December 31, 2020, with acute Achilles tendon ruptures (<4 weeks old) and were treated operatively by 56 different surgeons. The majority of the patients (n=114, 39.9%) were treated by a fellowship trained foot and ankle surgeon. Fellowship trained sports medicine surgeons, podiatrists, and all other orthopedic surgeons (general orthopedic surgeons, trauma trained, and total joint surgeons) treated a total of 85 (29.7%), 30 (10.8%), and 57 (19.9%) patients, respectively. Data did not specify inpatient versus outpatient procedures. A query was performed using current procedural terminology (CPT) codes to identify all patients with codes for primary Achilles tendon repairs (27,650), primary Achilles tendon repairs with grafts (27,652), secondary Achilles tendon repair with or without graft (27,654), tendon transfers (27,691), and unlisted/unspecified tendon procedures of the lower extremity (27,899). Individual charts were then checked by means of outpatient notes and operative reports. Inclusion data were injuries treated with primary end-to-end repairs, augmented repairs with flexor hallucis longus (FHL) transfers, augmented repairs with graft, augmented repairs with both FHL transfers and graft use, and isolated FHL transfers. The repair technique was at the discretion of the surgeon based on preoperative and intraoperative assessment of the patient. There was no standardized algorithm used in the determination of operatively treated patients. Demographics and tear locations (assessed by a combination of either preoperative magnetic resonance imaging (MRI) or by operative reports) were collected and analyzed (Table 1). Preoperative MRI was not used in all patients within the cohort as decided by the treating surgeon. Midsubstance tear of the tendon was defined as 2-6 cm from the calcaneal insertion, while distal insertional tears include distal avulsion tears and tears within 2 cm from the insertion site. Myotendinous tears occur at the myotendinous junction. All chronic tendon ruptures (>4 weeks old) were excluded from the study (n=27). During the study period, a total of 286 patients underwent operative treatment for an Achilles tendon rupture across our institution. Categorical variables were evaluated using chi-squared tests, z-tests for proportions, and continuous variables using Mann-Whitney U tests, and analyses of variances. Where applicable, Benjamini-Hochberg corrections were applied for multiple comparisons. A two-tailed p-value less than 0.05 was considered statistically significant. All statistical analyses were performed using Statistical Package for the Social Sciences version 26 (International Business Machines Corporation, Armonk, NY, USA).

**Table 1:** Tear Locations in Relation to Demographics and Operative Technique MTJ: myotendinous junction; BMI: body mass index; FHL: flexor hallucis longus tendon; *: comparative analysis not done; ^: pairwise comparison between distal and midsubstance tears

		Tear location						p-value
		Distal (N=35)		Midsubstance (N=237)		MTJ (N=14)		
		Mean/count	SD/%	Mean/count	SD/%	Mean/count	SD/%	
Age		53.3	12.5	41.6	13.7	35.1	7.4	<0.001
BMI, kg/m^2^		32.22	6.63	28.57	4.66	28.58	5.28	<0.001
Sex	Female	12	34.3	40	16.9	2	14.3	0.044
	Male	23	65.7	197	83.1	12	85.7	
Diabetes	Yes	7	20	17	7.2	1	7.1	0.042
Smoker	Yes	5	14.3	34	14.3	1	7.1	0.751
Chronicity	Acute	21	60	224	94.5	14	100	<0.001
Operative technique	FHL transfer only	8	22.9	0	0	0	0	*
	Standard end-to-end repair	12	34.3	183	77.2	8	57.1	<0.001
	Augmented repair	15	42.9	54	22.8	6	42.9	0.033
Augmented repair	Repair+FHL transfer	11	73.3	13	24.1	0	0	<0.001^
	Repair+graft	4	26.7	34	63	6	100	0.012^
	Repair+FHL transfer+graft	0	0	7	13	0	0	*

## Results

From 2017 to 2020, a total of 286 patients underwent operative treatment for an Achilles tendon rupture across our institution. Midsubstance tears were the most common tear location occurring in 237 of 286 (82.9%) patients. Distal insertional tears of the Achilles tendon were treated in 35 (12.2%) patients, and 14 (4.9%) patients had a tear at the myotendinous junction.

We found that patients with distal insertional tears had a significantly higher average age (53.3±12.5) than patients presenting with myotendinous tear locations (average age: 35.1±7.4; p<0.001). The average BMI was significantly higher in patients with distal insertional tears (average BMI: 32.2±6.6) compared to midsubstance tears (28.5±4.6; p<0.001) and proximal tears (28.5±5.3; p<0.001) (Table 1). There was a higher percentage of diabetic patients who underwent operative treatment for distal insertional tears (20%) compared to midsubstance tears (7.2%) (p<0.043). Regarding smoking status, there were no significant differences among tear locations.

Midsubstance tears were commonly treated with a standard end-to-end repair (n=183, 77.2%), but this technique was only utilized in 34.3% (n=12) of distal insertional and 57.1% (n=8) of myotendinous junction tears (Table 1). All isolated FHL transfers were performed for distal insertional tears by foot and ankle fellowship trained surgeons (n=8).

Augmented repairs (defined as any procedure using an isolated FHL transfer, FHL transfer plus end-to-end repair, or allograft use) were performed significantly more for distal insertional tears (42.9%, n=15) than for midsubstance tears (22.8%, n=54) (p<0.001) (Table 1).

## Discussion

Achilles tendon ruptures are among the most common tendon injuries seen by orthopedic surgeons. Surgeons are seeing increasing rates of ruptures, especially within the aging population. The aim of our research was to examine the relationship between patient demographics and tear location. Additionally, we sought to make note of the surgical technique used for each tear location in the operatively treated Achilles tendon rupture. We found that patients with advanced age and higher BMI presented with significantly more distal insertional tears (p<0.001), while younger patients had significantly higher rates of myotendinous tears (p<0.001). Regarding surgical technique, augmented repairs (defined as any procedure using an isolated FHL transfer, FHL transfer plus end-to-end repair, or allograft use) were performed significantly more often for distal insertional tears than for midsubstance tears (p<0.001).

Prior research is lacking as regards ATRs and demographics; however, prior literature coincides with our results. There is ample evidence supporting the increased risk of Achilles degeneration with increasing age [31,32]. The study by Maffulli found that increasing age leads to chronic degeneration of the tendon including decreased blood flow and a decrease in the tensile strength of collagen within the tendon [25]. Similarly, Ruan et al. demonstrated that the elasticity of a healthy Achilles tendon decreases with increasing age [32]. Although previous literature finds older patients, in general, to be at higher risk for tendon degeneration and tears, we found only such patients to have a higher rate of distal insertion tears, rather than all locations. Data supporting the increased risk of distal Achilles rupture with increasing BMI is sparse. A recent study by Macchi et al. showed that obesity was associated with a greater risk of upper extremity tendon tear and rupture [30]. They also noted that there was an increased risk of tendinopathy in both the upper and lower extremities [30]. Further literature exploring the role of BMI specifically in distal Achilles ruptures is therefore warranted. Lastly, the 2-6 cm region proximal to the Achilles tendon’s calcaneal insertion (midsubstance) is the most common rupture location [33], which agrees with our findings. This region has been found to have a small cross-sectional area, large eccentric loads, and hypovascularity, all predisposing this area to rupture [33,34].

In contrast to our findings, some literature demonstrates a lack of relationship between certain demographic factors and ATRs. Noback et al. reported no significant increase in ATRs with an increase in BMI, with a mean BMI of 27.77 in the rupture group and 26.66 in their control [35]. Similarly, a review by Claessen et al. found limited evidence to correlate increased BMI and higher ATR rates [36]. In a review by Macchi et al., they noted that although there was a significant increase in Achilles tendinopathy with obesity, they found no association between BMI and Achilles rupture [30]. However, to our knowledge, there have been no studies that correlated demographics with the location of Achilles rupture, which makes our study unique in this regard.

Although augmentation techniques are classically used for chronic tears, we found that our cohort also used augmentation techniques for acute tears. In the acute setting, augmentation can be used in patients with severe degeneration of the tendon, such as those who had a history of Achilles tendonitis [37]. Augmentation can provide a stronger fixation construct for weaker tissue, such as with FHL or FDL transfer.

Given our findings of older patients with higher BMI having distal tears, this brings to question the utility of preoperative MRI. The debate on the use of advanced imaging for the management of ATRs remains controversial in the literature. The American Academy of Orthopaedic Surgeons’ clinical practice guideline recommendation was inconclusive regarding the routine use of MRI for diagnosing acute Achilles ruptures owing to the lack of evidence in the literature to support its use [12,38-40]. Khan et al. previously reported MRI sensitivity of 94% and a specificity of 56% in the evaluation of ATR [34]. Although routine preoperative imaging with magnetic resonance imaging (MRI) may be commonplace for many treating surgeons, previous literature has also claimed its unnecessary use for diagnosis [41]. Nonetheless, several authors discourage the routine use of preoperative MRI, since it is too expensive and a time-wasting imaging modality [42]. Additionally, authors have argued that MRI is often unavailable within a short time, thus leading to an unjustified delay in surgery [41]. Within our study cohort, patients >50 years old and BMI > 30 kg/m^2^ were more likely to have distal insertional Achilles tendon ruptures and also were more likely to require the use of augmentation. Perhaps these patients would benefit from a preoperative MRI to determine tear location and the possible necessity for a more complex repair.

We note some limitations to our study, including its retrospective nature and small sample size. We limited our study population to acute ATRs only (<4 weeks), therefore lacking an evaluation of chronic presentations. Future similar studies, comparing both acute and chronic tears, may help better define the role of demographics in both patient populations. Although we chose to strictly examine operatively treated Achilles tendon ruptures, nonoperative management of such injuries is becoming more and more popular, as research has shown positive outcomes in nonoperatively treated patients [43]. Further research should include both nonoperatively and operatively treated patients. Lastly, our study did not take into account preoperative MRI findings for all patients within our cohort or the rate of MRI use for preoperative planning. Future analyses examining the role and frequency of preoperative MRI use in Achilles tendon ruptures would be helpful in further exploring this.

## Conclusions

The findings of our study suggest that a subset of patients, particularly advanced age and higher BMI, are more likely to present with distal Achilles tendon ruptures. Additionally, patients who had distal tears were more likely to require an augmented repair technique. In such patients, the role of preoperative imaging may be particularly helpful with operative planning. Our results highlight the need for future research to further define the relationship between increasing age and higher BMI patients sustaining distal tears more often than midsubstance tears. With increasing BMI among the general public and older patients experiencing Achilles tendon ruptures more frequently, this combination of patient demographics will continue to be seen within the orthopedic community. This also highlights the need for further research regarding the cost-benefit analysis for the use of preoperative MRI in these patient populations.
